# Quantitative Comparison of Commercial and Non-Commercial Metal Artifact Reduction Techniques in Computed Tomography

**DOI:** 10.1371/journal.pone.0127932

**Published:** 2015-06-01

**Authors:** Dirk Wagenaar, Emiel R. van der Graaf, Arjen van der Schaaf, Marcel J. W. Greuter

**Affiliations:** 1 Department of Radiation Oncology, University Medical Center Groningen, University of Groningen, Groningen, The Netherlands; 2 KVI-Center for Advanced Radiation Technology, University of Groningen, Groningen, The Netherlands; 3 Department of Radiology, University Medical Center Groningen, University of Groningen, Groningen, The Netherlands; Northwestern University Feinberg School of Medicine, UNITED STATES

## Abstract

**Objectives:**

Typical streak artifacts known as metal artifacts occur in the presence of strongly attenuating materials in computed tomography (CT). Recently, vendors have started offering metal artifact reduction (MAR) techniques. In addition, a MAR technique called the metal deletion technique (MDT) is freely available and able to reduce metal artifacts using reconstructed images. Although a comparison of the MDT to other MAR techniques exists, a comparison of commercially available MAR techniques is lacking. The aim of this study was therefore to quantify the difference in effectiveness of the currently available MAR techniques of different scanners and the MDT technique.

**Materials and Methods:**

Three vendors were asked to use their preferential CT scanner for applying their MAR techniques. The scans were performed on a Philips Brilliance ICT 256 (S1), a GE Discovery CT 750 HD (S2) and a Siemens Somatom Definition AS Open (S3). The scans were made using an anthropomorphic head and neck phantom (Kyoto Kagaku, Japan). Three amalgam dental implants were constructed and inserted between the phantom’s teeth. The average absolute error (AAE) was calculated for all reconstructions in the proximity of the amalgam implants.

**Results:**

The commercial techniques reduced the AAE by 22.0±1.6%, 16.2±2.6% and 3.3±0.7% for S1 to S3 respectively. After applying the MDT to uncorrected scans of each scanner the AAE was reduced by 26.1±2.3%, 27.9±1.0% and 28.8±0.5% respectively. The difference in efficiency between the commercial techniques and the MDT was statistically significant for S2 (p=0.004) and S3 (p<0.001), but not for S1 (p=0.63).

**Conclusions:**

The effectiveness of MAR differs between vendors. S1 performed slightly better than S2 and both performed better than S3. Furthermore, for our phantom and outcome measure the MDT was more effective than the commercial MAR technique on all scanners.

## Introduction

Metal implants like prostheses, plates and screws are routinely used in bone surgery. In addition, amalgam (a mercury alloy) is commonly present in tooth fillings. The presence of these metal objects causes reconstructed images from computed tomography (CT) to be suboptimal. Typical streaking artifacts, known as metal artifacts, occur in the presence of strongly attenuating objects and are caused by photon starvation, scattering, beam hardening and other effects[1].

Filtered back projection is the most commonly used CT reconstruction algorithm. Filtered back projection yields good results in ideal conditions but in the presence of metal implants metal artifacts can become so severe that the reconstructed images diagnostic accuracy is seriously hampered[2]. Furthermore, accurate CT values are crucial for attenuation correction in PET-CT scanning[3,4], dose calculation in X-ray radiotherapy planning[5] and stopping power calculation in proton therapy planning[6,7].

In the previous ten years many metal artifact reduction (MAR) techniques have been extensively developed, most of which use a technique of replacing the affected projections in sinogram space[8–13], although other techniques have been reported[14]. Several studies have compared the effectiveness of these techniques[15–20].

Recently, vendors have started offering their MAR techniques for their scanners commercially. In addition, Boas et al. developed a MAR technique called the metal deletion technique (MDT) which does not require sinogram data, but is able to reduce metal artifacts using reconstructed images, making it more practical to implement the technique clinically[19].

Although a comparison of the MDT to other MAR techniques exists[18], a comparison to commercially available MAR techniques is lacking.

The aim of this study was therefore to quantify the difference in effectiveness of the currently available MAR techniques of different scanners and the MDT technique.

## Materials and Methods

### Phantom description

**Fig 1:**
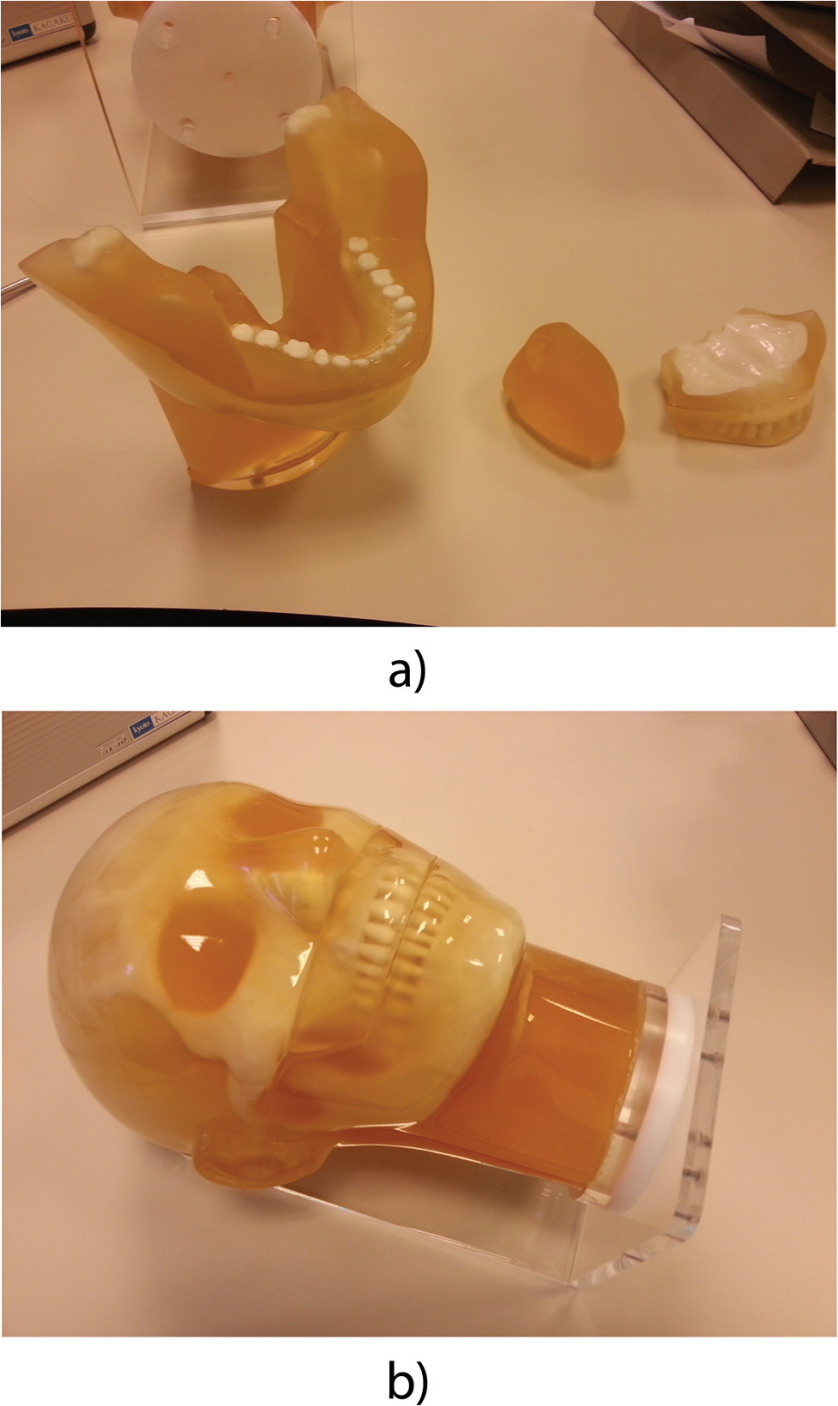
The Kyoto Kagaku PH-47 head/neck phantom. (a) The assembled phantom. (b) The disassembled jaw.

The scans were made using an anthropomorphic head and neck phantom (Kyoto Kagaku, Kyoto, Japan) (Fig 1A). The phantom is made of a soft tissue substitute (SZ-50) and an epoxy based resin containing hydroxyapatite for the teeth and bones. The phantom consists of four parts which can be disassembled: the upper jaw, the lower jaw including the anterior half of the neck, the tongue and the remainder of the head and neck (Fig 1B).

Three dental implants were constructed using dental grade amalgam. The size of each of the implants was approximately 150 mm^3^. To ensure that the implants were repeatedly placed in the same location between the teeth, a silicon mould was made of the teeth in which the implants were placed. This was the most realistic position we could place the implants without damaging the phantom.

### Scan protocol and data processing

Three vendors (Philips Healthcare, General Electric (GE) Healthcare and Siemens Healthcare) were asked to use their preferential CT scanner for applying their MAR techniques in a head and neck CT scan. To allow easy and accurate positioning in the CT scanners, the phantom was mounted on an L-shaped PMMA frame. Six scans were made alternating between a scan with and a scan without amalgam implants. The scans were performed on a Philips Brilliance ICT 256 (S1), a GE Discovery CT 750 HD (S2) and a Siemens Somatom Definition AS Open (S3). The acquisition and reconstruction parameters shown in Table 1 were chosen by the vendors for optimal MAR performance using a CTDI_vol_ of 9 mGy. For the reconstruction, the slice thickness was set to be minimal, which was 0.67 mm, 0.625 mm and 0.6 mm for S1, S2 and S3 respectively.

**Table 1:** Scan protocol for the CT scans of the three scanners.

	S1	S2	S3
**Acquisition parameters**			
Scan mode	Spiral	Spiral	Spiral
Tube voltage (kV)	120	140/80*	120 + 140/80*
Automatic Exposure Control	Yes	no	No
Exposure (mAs)	53–129	168	101
Average CTDIvol (mGy)	9.36	9.66	9.02
Rotation time (s)	0.4	0.6	0.5
Collimation (mm)	64 x 0.625	64 x 0.625	20 x 0.2
Pitch factor	0.671	0.984	1.2
**Reconstruction parameters**			
MAR technique	O-MAR	SMAR	MARIS
Slice thickness (mm)	0.67	0.625	0.6
Reconstruction Index (mm)	0.67	0.625	0.6
Number of slices	366	399	409
Field of view (cm)	25	25	25

S1: Philips Brilliance ICT 256, S2: GE Discovery CT 750 HD, S3: Siemens Somatom Definition AS Open, O-MAR: Metal Artifact Reduction for Orthopedic Implants, SMAR: Smart Metal Artifact Reduction, MARIS: Metal Artifact Reduction in Image Space.

* S2 scanned using dual energy. S3 used both a metal artifact reduction technique based on single and on dual energy using a tube voltage of 140/80 kV.

The MAR technique used on S1 was MAR for Orthopedic Implants (O-MAR). Smart MAR (SMAR) was used on S2 and S3 used MARIS, a MAR technique which creates five different reconstructions (MAR0-MAR4) from which the best image was chosen. In addition S3 used a dual energy based technique. The exact functioning of the commercial MAR techniques was not available. In addition to each scanner’s own MAR technique, the reconstructed scans with metal artifacts were reconstructed using the MDT technique.

A rigid registration was then performed using the Mirada RTx software package (Mirada Medical UK, Oxford, UK) to compensate for movement of the phantom between scans.

### Quantitative comparison

To quantify the effectiveness of the presence of metal artifacts, the average absolute error (AAE) was calculated for each reconstruction:

$$AAE\;=\;\frac{1}{N}\sum_{i\;=\;1}^{N}\frac{1}{3}\sum_{j=1}^{3}\left|x_{i}-t_{i,j}\right|$$

Where N is the number of voxels and x_i_ and t_i,j_ are the measured CT values at the *i*-th voxel and its value obtained from the *j*-th reference image respectively. When calculating the AAE between reference scans, all three reference scans were compared to each other.

As we were mainly interested in the reduction of streaking artefacts we only considered voxels that were not too far or too close to the metal implants. To achieve this, voxels were excluded if they were outside a 170 x 134 mm^2^ rectangle containing the entire phantom or within a square around a metal implant with a margin of 1 mm.

**Fig 2:**
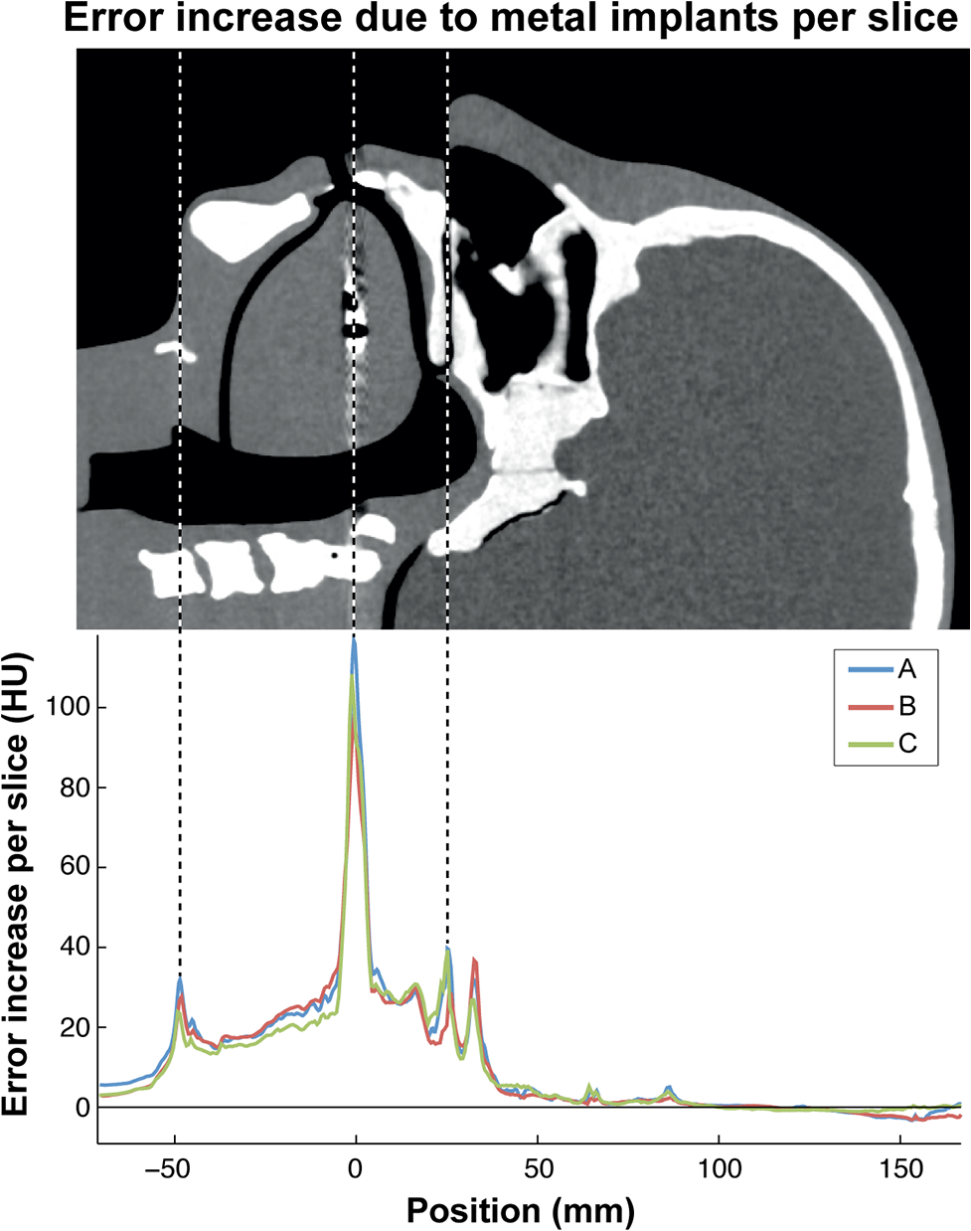
The increase in average absolute error (AAE) of all standard CT reconstructions of the phantom containing three amalgam implants as a function of the position of the slice. A high error was observed in a 9 mm wide region around the amalgam implants at 0 mm. In addition, a high error was observed at -48 mm and +24 mm which were caused by movement of the tongue and the upperjaw due to the insertion of amalgam implants respectively. S1: Philips Brilliance ICT 256, S2: GE Discovery CT 750 HD, S3: Siemens Somatom Definition AS Open.

The increase in AAE per slice was calculated for all scans made with metal implants without MAR and plotted as a function of the slice position (Fig 2). This was calculated by subtracting the average AAE between two reference scans from the average AAE of a standard reconstruction of a scan with metal implants. A strong increase in AAE was observed at the position of the metal implants. Only the 9 mm of slices in which metal artifacts were most strongly contributing to the AAE per slice was used in the following calculations of the AAE.

### Statistical analysis

Standard errors were calculated for the AAE of each reconstruction technique. A Student t-test was performed to evaluate the difference in AAE between different MAR techniques. All tests were performed in the Matlab R2014a package and all p-values were two-tailed and considered statistically significant if p ≤ 0.05.

## Results

**Fig 3:**
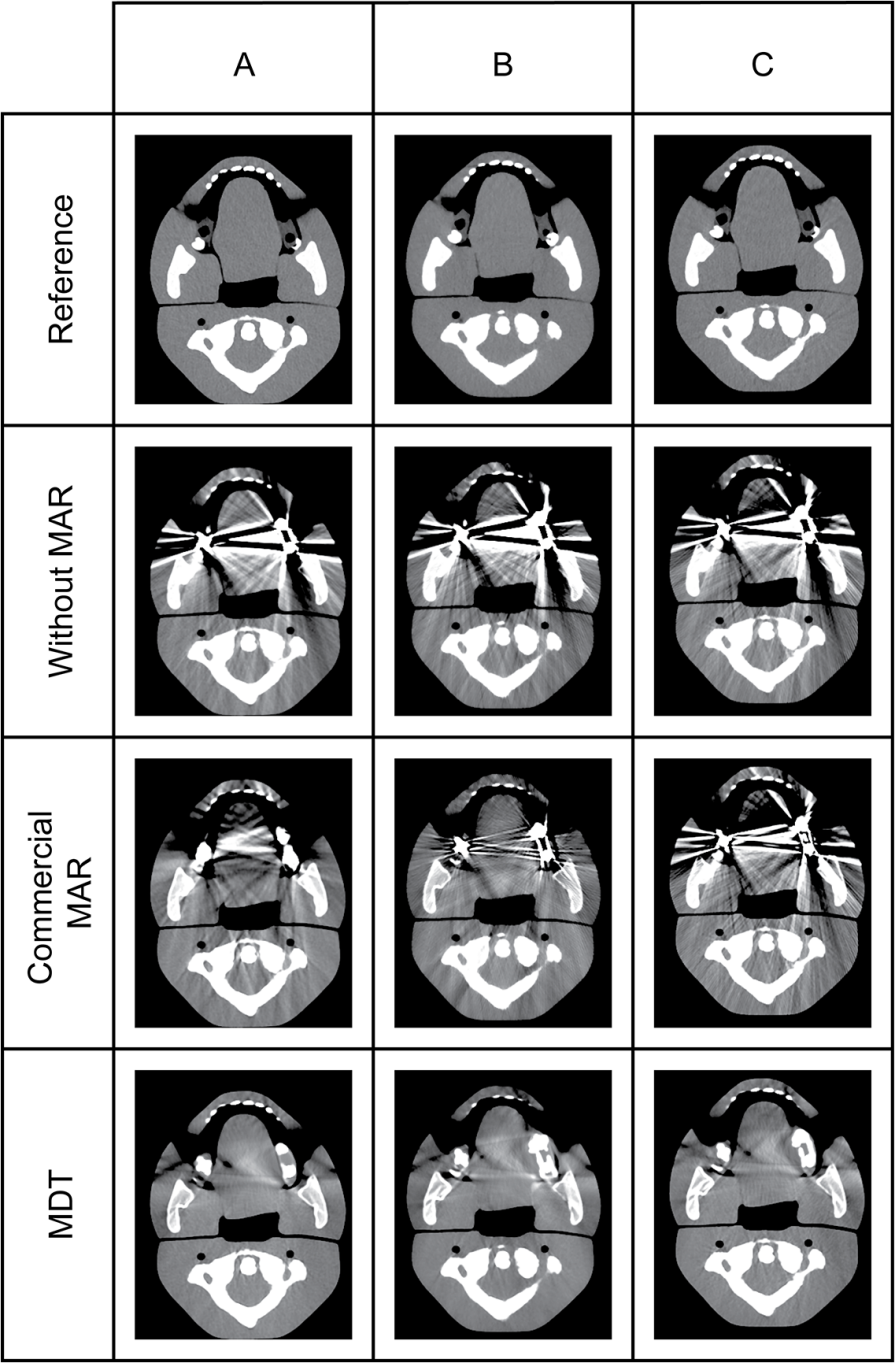
CT reconstructions made with and without metal implants and with different MAR techniques. For S3, the reconstruction using MAR2 is displayed. The window and level were set at 400 and 40 respectively. S1: Philips Brilliance ICT 256, S2: GE Discovery CT 750 HD, S3: Siemens Somatom Definition AS Open.

**Fig 4:**
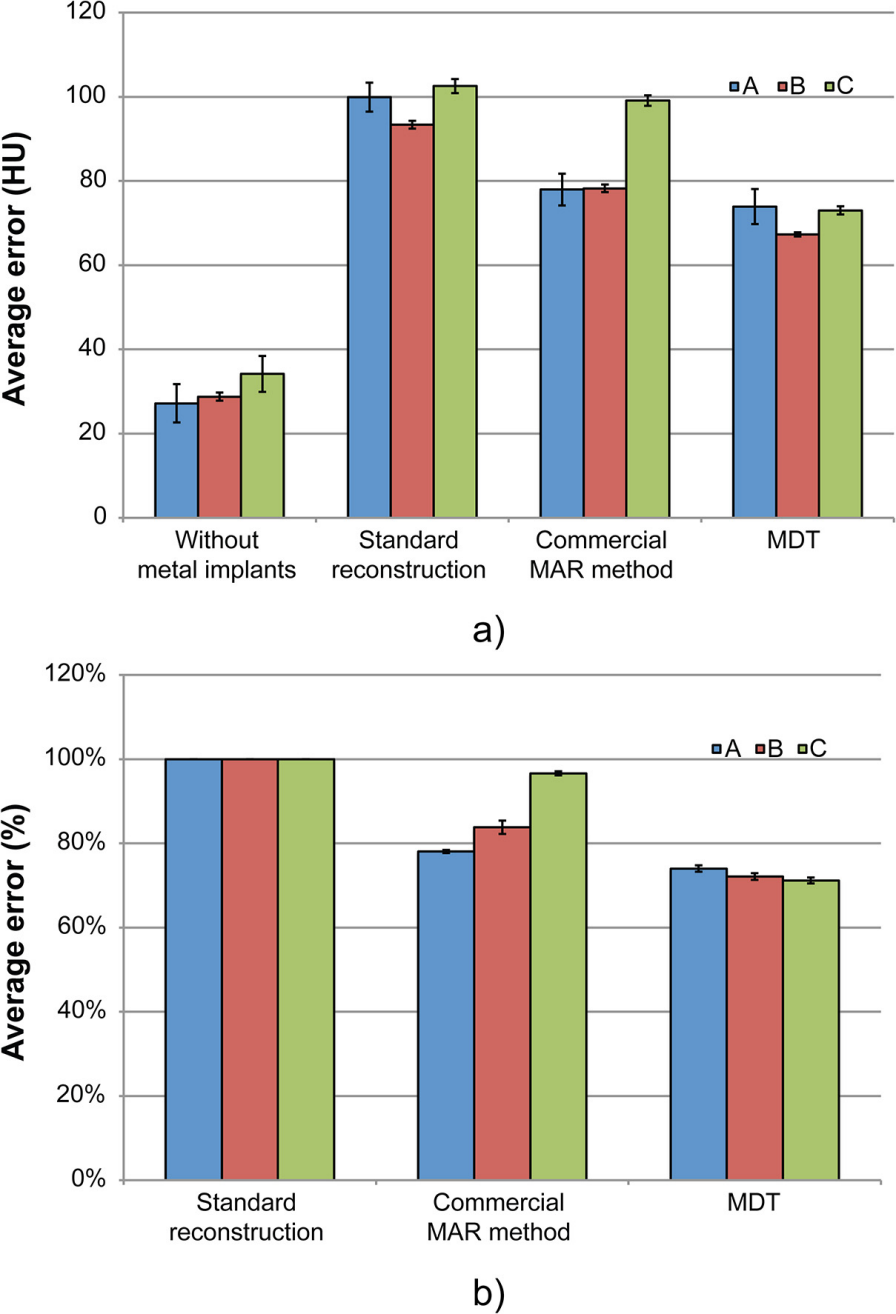
The average absolute error (AAE) with and without different metal artifact reduction (MAR) techniques. (a) The AAE between reference scans, for scans without MAR, for scans with the commercial MAR technique and for scans corrected using the MDT software. For S3, MAR2 is displayed. (b) The AAE reduction after the application of both the commercial MAR techniques and the metal deletion technique (MDT). S1: Philips Brilliance ICT 256, S2: GE Discovery CT 750 HD, S3: Siemens Somatom Definition AS Open.

The reconstructions of scans of the phantom with metal implants before and after MAR for each scanner is shown in Fig 3. For each scanner, all scans were made sucessfully. For S1, one scan with metal implants was corrupt and could not be opened and was excluded from the analysis. The AAE was determined for all reconstructions and is shown in Fig 4A.

For the different techniques offered on S3, the AAE depended on the MAR intensity and was found to be optimal for MAR2 (AAE = 100.69 HU; 99.79 HU; 99.09 HU; 99.63 HU; 101.75 HU, respectively for MAR0-4). The dual energy technique available on S3 yielded an increase in AAE (AAE = 114.52 HU). S1 and S2 used only one MAR technique.

When using the commercial MAR techniques, S1 performed best (AAE = 78.0±3.8 HU (one standard error)) followed closely by S2 (AAE = 78.3±0.9 HU). Finally S3 had the least effective MAR technique (AAE = 99.1±1.2 HU).

The MDT technique reduced the AAE more than the commercial MAR technique for all scanners as shown in Fig 4B. The commercial techniques reduced the AAE by 22.0±1.6%, 16.2±2.6% and 3.3±0.7% for S1, S2 and S3 respectively. After applying the MDT to uncorrected scans of each scanner the AAE was reduced by 26.1±2.3%, 27.9±1.0% and 28.8±0.5% for S1, S2 and S3 respectively. The difference in efficiency between the commercial technique and the MDT was statistically significant for S2 (p = 0.004) and S3 (p<0.001), but not for S1 (p = 0.63).

## Discussion

The effectiveness of MAR differs between the scanners. S1 performed slightly better than S2 and both performed better than S3. Furthermore, for all scanners the MDT was more effective than the commercial MAR technique which was statistically significant for S2 and S3.

S3 had the least effective MAR technique in this test and also has a different collimation setting on their scanner. It is hard to quantify the effect of this on MAR effectiveness. However, this scanner was chosen by Siemens as their preferential scanner for a MAR demonstration. Moreover, from a follow-up discussion with Siemens it was concluded that the results were in agreement with their expectations. This is further supported by the fact that the MDT software was able to acquire good results on the same scanner, indicating that the poor results are due to the difference in MAR technique.

Several studies compared different published MAR techniques[15–20]. Mouton et al. performed a simulation study in which a digital image was used to calculate a sinogram from which different reconstructions were made using 12 different MAR techniques[15]. The performance of each algorithm was compared by comparing its root mean squared error to a filtered backprojection reconstruction. The results showed a 17–80% reduction in root mean squared error. Our study also found large differences in MAR effectiveness. Kidoh et al. evaluated the improvement in image quality for dental MAR using the MAR technique by Philips. The quality of the images as evaluated by two radiologists was found to improve and the image noise was found to decrease (p < 0.01)[20]. No studies evaluated the MAR techniques used by GE and Siemens. Lastly, Golden et al. compared different MAR techniques, including the MDT and also found MDT to be the most effective, although the technique was not compared to commercial techniques[18].

The MDT method performs better in this phantom experiment than the commercial available methods. However, this research was based on a quantitative analysis of the average absolute error. Rinkel et al. found that the contrast to noise ratio is independently affected by metal artefacts and can be used as a separate outcome measure[16]. Should an analysis of the contrast to noise ratio be included, the relative differences in performance between the different methods might change. In addition, the results we found are valid for this anthropomorphic head phantom, and might also change for other metal implants in other body parts or in an in-vivo situation.

This is the first study to compare commercially available MAR techniques offered on three scanners using their recommended scanner, acquisition and reconstruction parameters and a non-commercial technique. The use of advanced registration software minimized the error due to movement and morphing of the phantom. Furthermore, the use of an anthropomorphic phantom allowed both the insertion and removal of metal implants while creating a dataset close to the clinical situation.

There were two limitations of this study. Firstly, the acquisition parameters were different for the different scanners. Using identical acquisition parameters for all scanners would yield an unfair comparison since each CT scanner has different optimal settings for its MAR technique. In this study we therefore used the vendors recommended set of acquisition and reconstruction parameters, at approximately the same dose. Secondly, the contrast to noise ratio was not considered. More research using a dedicated phantom would be necessary to evaluate this.

The effectiveness of MAR differs between vendors. S1 performed slightly better than S2 and both performed better than S3. Furthermore, for our phantom and outcome measure the MDT was more effective than the commercial MAR technique on all scanners.
